# Identification of Cancer-Associated Fibroblast Subtype of Triple-Negative Breast Cancer

**DOI:** 10.1155/2022/6452636

**Published:** 2022-04-23

**Authors:** Maoli Wang, Ruifa Feng, Zihao Chen, Wenjie Shi, Cheng Li, Huiquan Liu, Kejin Wu, Dajin Li, Xiqing Li

**Affiliations:** ^1^Department of Breast Surgery, Obstetrics and Gynecology Hospital, Fudan University, Shanghai 200090, China; ^2^Breast Center of the Second Affiliated Hospital of Guilin Medical University, 541100 Guilin, Guangxi, China; ^3^University Hospital for Gynecology, Pius-Hospital, University Medicine Oldenburg, 26121 Oldenburg, Germany; ^4^Department of Orthopaedic Surgery, Beijing Jishuitan Hospital, Fourth Clinical College of Peking University, 100035 Beijing, China; ^5^Department of Radiation Oncology, The First Affiliated Hospital of Anhui Medical University, 230032 Hefei, China; ^6^NHC Key Laboratory of Reproduction Regulation (Shanghai Institute of Planned Parenthood Research), Shanghai Key Laboratory of Female Reproductive Endocrine Related Diseases, Hospital of Obstetrics and Gynecology, Fudan University Shanghai Medical College, Shanghai 200080, China; ^7^Oncology Department, Henan Provincial People's Hospital, Zhengzhou University People's Hospital, 450003 Zhengzhou, China

## Abstract

**Background:**

There is limited knowledge about the role of cancer-associated fibroblasts (CAF) in the tumor microenvironment of triple-negative breast cancer (TNBC).

**Methods:**

Three hundred and thirty-five TNBC samples from four datasets were retrieved and analyzed. In order to determine the CAF subtype by combining gene expression profiles, an unsupervised clustering analysis was adopted. The prognosis, enriched pathways, immune cells, immune scores, and tumor purity were compared between CAF subtypes. The genes with the highest importance were selected by bioinformatics analysis. The machine learning model was built to predict the TNBC CAF subtype by these selected genes.

**Results:**

TNBC samples were classified into two CAF subtypes (CAF+ and CAF-). The CAF- subtype of TNBC was linked to the longer overall survival and more immune cells than the CAF+ subtype. CAF- and CAF+ were enriched in immune-related pathways and extracellular matrix pathways, respectively. Bioinformatics analysis identified 9 CAF subtype-related markers (ADAMTS12, AEBP1, COL10A1, COL11A1, CXCL11, CXCR6, EDNRA, EPPK1, and WNT7B). We constructed a robust random forest model using these 9 genes, and the area under the curve (AUC) value of the model was 0.921.

**Conclusion:**

The current study identified CAF subtypes based on gene expression profiles and found that CAF subtypes have significantly different overall survival, immune cells, and immunotherapy response rates.

## 1. Introduction

Breast cancer (BC) has been the most frequent carcinoma and the second cause of cancer death in women. There were more than 2.2 million patients diagnosed with BC and approximately 0.7 million deaths caused by BC in 2020 [1]. BC is a heterogeneous disease that includes triple-negative BC (TNBC) and nontriple-negative BC (NTNBC). The absence of estrogen receptors (ER), progesterone receptors (PR), and human epidermal growth factor receptor 2 (HER2) is the characteristic of TNBC (15% to 20% of BC samples) [2]. TNBC patients have a worse 5-year survival rate than those with other types of BC. For example, 30% of them could not survive 5 years after diagnosis [3]. Patients with TNBC are treated mainly with chemotherapy, and there are no targeted therapies available for them [4]. There is an urgent need for developing new therapies for TNBC patients.

Recent studies suggest that the tumor microenvironment (TME) exerts critical functions in tumor growth and progression control. TME is composed of cancer cells, as well as supporting cells such as stromal cells and infiltrating immune cells [5]. In multiple solid tumor types, cancer-associated fibroblasts (CAFs) are found as one of the most prevalent stromal cells [6]. CAFs consist of quiescent CAFs (qCAFs), tumor-restraining CAFs (rCAFs), and tumor-promoting CAFs (pCAFs) [7]. Among these three types of CAFs, qCAFs and rCAFs are typically found in low-stage cancers, and pCAFs are detected in advanced-stage cancers. A body of research indicates that CAFs play a crucial role in a variety of protumorigenic biological processes, such as invasion of tumor cells, resistance to chemotherapy, and evasion of immune cells [8, 9]. For example, CAFs could contribute to tumor development by providing oxygen and suppressing the immune cells in the TME [10]. However, other studies suggest that CAFs can exert a tumor-suppressive impact on the TME [11]. For example, a previous study discovered that CAFs have a vital suppressive impact on fibrosarcoma [12]. The collection of these research endeavors embodies the importance that the effect of CAFs on TNBC prognosis should be clarified.

Immune checkpoint blockade (ICB) such as PDL1/PD1 antibodies has been linked to improved clinical outcomes in TNBC, making ICB an appealing treatment option for TNBC patients [13]. Progress-free survival (PFS) was considerably greater in the PD-1 antibody group (9.7 months) than in the control group (5.6 months) in a randomized, double-blind, phase III TNBC trial (NCT02819518) (*p* value = 0.0012) [14]. However, only 18.5 percent of TNBC samples from the KEYNOTE012 trial reacted to PD1/PDL1 antibodies, which is far from satisfactory [15]. According to the new research, TNBC is not a unique illness, and the identification of subgroups/subtypes within TNBC samples might contribute to finding the right patients for PD1/PDL1 antibodies [16].

Toward this purpose, we analyzed and compared CAF subtypes from the discovery datasets of TNBC samples, as well as disclosed their molecular and biological properties. In the training dataset, the CAF+ subtype was linked to poor prognosis. We then built a prediction model to predict CAF subtypes using a machine learning method based on 9 genes. The predicted CAF subtypes of samples from an independent breast cancer dataset showed that the CAF+ subtype had a poor clinical outcome. Results from ICB datasets also demonstrated that the CAF subtypes have a crucial effect on TNBC resistance to ICB.

## 2. Materials and Methods

### 2.1. Patients and Specimens

Four TNBC datasets and 335 samples were utilized as discovery datasets for CAF subtype classification. These four datasets came from The Cancer Genome Atlas (TCGA) (https://portal.gdc.cancer.gov/) and the Gene Expression Omnibus (GEO) (https://www.ncbi.nlm.nih.gov/geo/). The discovery datasets included GSE19615 (28 TNBC samples) [17], GSE21653 (84 TNBC samples) [18], GSE58812 (107 TNBC samples) [19], and TCGA (116 TNBC samples). Based on the R GEOquery package [20], the normalized expression profiles of GSE19615, GSE21653, and GSE58812 were retrieved from the GEO website by the accession numbers. The TCGA-TNBC dataset's level 3 raw count expression profiles were retrieved using the ‘TCGAbiolinks' R package [21]. The dates for downloading expression profiles from the TCGA and GEO datasets were September 20, 2021 and September 27, 2021. The created fibroblast subtype was verified using an independent breast cancer dataset (the METABRIC dataset) [22]. 313 ER-negative and HER2-negative breast cancers with obtainable overall survival (OS) information and gene expression matrix were retrieved from METABRIC [16].

The link between CAF subtypes and ICB response was assessed using three different datasets (GSE78220 [23], GSE35640 [24], and IMvigor210 [25]) comprising patients treated with ICB. GSE78220 contains pretreatment mRNA expression data from anti-PD-1 therapy in 28 melanoma samples. GSE35640 contains pretreatment mRNA expression data from MAGE-A3 immunotherapeutic therapy in 65 melanoma and lung cancer samples. IMvigor210 contains pretreatment mRNA expression data from anti-PD-L1 therapy in 348 cancer samples.

### 2.2. Batch Effect Correction and Consensus Clustering (CC) Analysis

Using the gene set variation analysis (GSVA) R program, the expression profiles of GSE19615, GSE21653, GSE58812, and TCGA-TNBC were converted into the matrix of CAF gene sets. CAF related biomarkers and gene sets were summarized from studies and listed in Supplementary Table 1 [26–28]. The batch effect was shown using principal component analysis (PCA) before and after the conversion. The consensus clustering algorithm from the R ‘ConsensusClusterPlus' package was used to determine the probable CAF subtypes by the expression matrix of CAF gene sets [29]. The optimal cluster number for the consensus clustering algorithm was chosen based on the tracking plot, delta area, the average silhouette width value, and CDF results [30].

### 2.3. Single-Sample Gene Set Enrichment Analysis (ssGSEA) and ESTIMATE

In the supplementary data from Bindea's study, the gene sets corresponding to immune cells were obtained [31]. By applying the ssGSEA method from the GSVA package, the enrichment scores of 28 immune cells for the TNBC sample were measured by the gene expression matrix. By the ESTIMATE algorithm, stromal, immune scores, and tumor purity were computed by the gene expression matrix. The values of stromal, immune scores, and tumor purity were then normalized of ‘min-max normalization.' Min-max normalization is one of the most frequently used methods for data normalization. The minimum value of stromal, immune scores, and tumor purity was converted into 0, the highest value was converted into 1, and other values were then transformed into a value range from 0 to 1. Our next step was to compare the differences between the different CAF subtypes by Student's *t*-test.

### 2.4. Differentially Expressed Gene (DEG) Screening and Enrichment Analysis

In order to select the key genes among the two CAF subtypes, we used the DEG analysis. Packages, including ‘limma,' ‘edgeR,' and ‘DESeq2,' are the most popular and accurate methods for DEG analysis. The principles and the preferred data for these three DEG methods are different. The linear model is adopted in the ‘limma' package, but ‘edgeR' and ‘DESeq2' packages calculated the DEGs by the negative binomial distribution [32]. The differential expression test for ‘edgeR' and ‘DESeq2' are exact test, and the differential expression test for ‘limma' is empirical Bayes method. Besides, the input data for ‘edgeR' and ‘limma' must be the expression profiles after the normalization. For the datasets with a small number of replicates, ‘limma' is the safest choice [33]. We do not use DESeq2 to obtain the DEGs among the two CAF subtypes because more computer resources and time are needed in the process of calculation [33]. Since the samples from GEO datasets are smaller in GSE19615, GSE21653, and GSE58812, the DEGs were analyzed using the R package “limma” [34]. In the TCGA-TNBC dataset, which contains more samples, “edgeR” package was used to determine the DEGs between two subtypes [35]. The DEGs with *p* value < 0.05 and ∣log2(foldchange) | >0.5 for each dataset were then filtered.

The robust rank aggregation (RRA) approach, which can decrease dataset bias, was utilized to combine the filtered DEGs from the above four expression datasets. The RRA approach is based on the assumption that a gene will be considered a robust DEG if it ranks first in all of the DEG gene lists. RRA computed significance scores for all genes, and only the statistically important genes were kept. To get robust DEGs among diverse datasets, RRA was used using the “RobustRankAggreg” package in R language [36]. The DEGs were selected by the cutoff of ∣log2(foldchange) | >0.5 and *p* value < 0.05. Then, functional Gene Ontology (GO), Kyoto Encyclopedia of Genes and Genomes (KEGG), and Reactome enrichment analyses were conducted. Using the OS information, the differences of DEGs survival curves were calculated. And the prognostic-related genes were selected by the cutoff of *p* value < 0.05, and the Kaplan–Meier model was conducted to illustrate the difference between survival curves.

### 2.5. CAF Subtype Prediction Model

Using random forest (RF), decision tree (DT), and *k*-nearest neighbors (KNN) approaches from the R package “caret,” we constructed CAF subtype predictors. The package ‘caret' is a prevalent application for building prediction models and contains many prevalent machine learning approaches [16]. During the model training process, prognostic-related genes expression data were utilized. In the first step, the expression data was randomly divided into the training dataset (50 percent) and the testing dataset (50 percent). Afterward, the parameter search accompanied by the fivefold cross-validation procedure was applied. We compared the prediction accuracy of machine learning models, and the machine learning model with the highest value of area under the curve (AUC) was selected. Then, the genes with the highest importance were kept in model construction. The testing dataset was then used to assess the developed model's ability to predict. Finally, the CAF subtypes of samples from the METABRIC dataset were predicted by the constructed model, and the METABRIC dataset was used as an independent validation dataset to confirm the CAF subtypes and prognosis association.

### 2.6. Protein Expression Profiles of Selected Genes in the Human Protein Atlas (HPA)

The protein values of hub genes were calculated based on the data from HPA data. Immunohistochemistry (IHC) staining was represented by a number: not detected/negative (0), low (1), medium (2), and high (3). The IHC intensity was represented by a number: none/negative (0), weak (1), moderate (2), and strong (3). The IHC quantity was represented by a number: none/negative (0), <25% (1), 25–75% (2), and >75% (3). The IHC score was determined by the sum of staining intensity and the staining quantity.

### 2.7. Statistical Analysis

R language was used to implement the statistical analysis. For the purpose of examining the differences between two groups, Student's *t*-test was implemented. If not stated otherwise, *p* values less than 0.05 were considered significant.

## 3. Results

### 3.1. CAF Subtypes with Distinct Survival Rates

**Figure 1:**
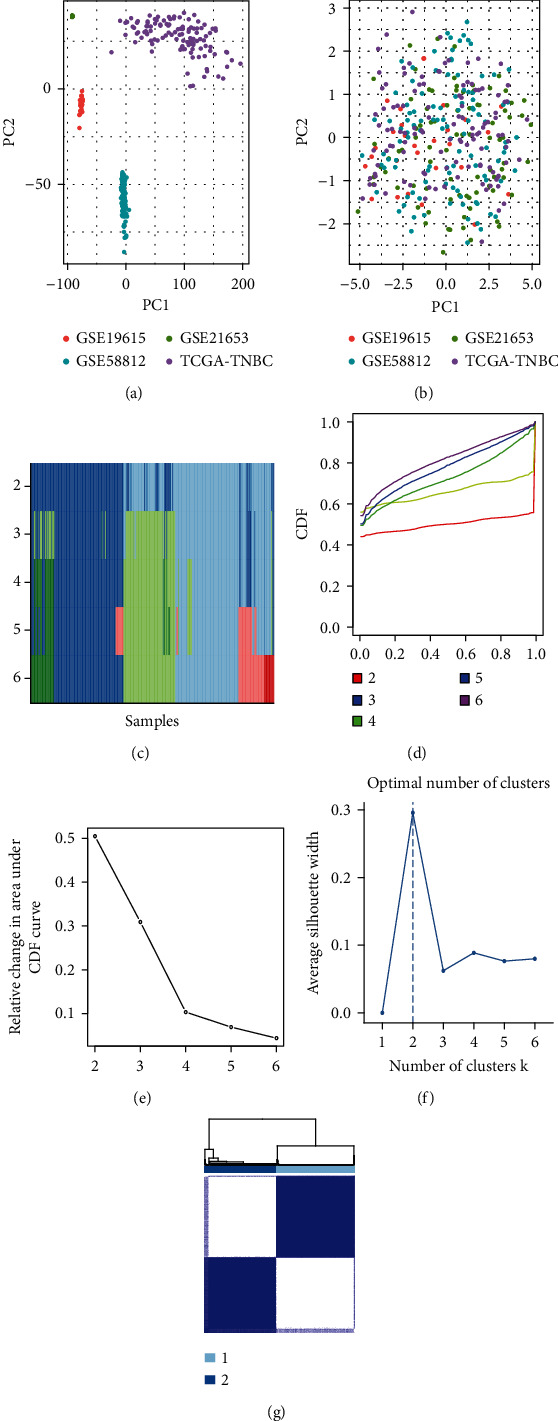
Fixing batch effects and selecting the optimum number of cancer-associated fibroblast (CAF) subtypes. (a) The differences among samples obtained from different datasets are illustrated via principal component analysis (PCA) before the removal of batch effects. (b) The differences among samples obtained from different datasets are reduced after the removal of batch effects. (c) Tracking plot for *k* = 2 to 6. The tracking plot shows the consensus cluster of TNBC samples (in columns) at each *k* (in rows). Promiscuous samples are identified and plotted with this plot to identify weak class membership and to visualize the distribution of cluster sizes across *k*. (d) The empirical cumulative distribution function (CDF) plot displays the consensus distributions of *k*. (e) Relative change under area CDF plots for each *k*. These two plots are used to find the *k* at which the distribution reaches an approximate maximum stability. An optimal *k* is determined by the *k* value at which CDF reaches its maximum or the *k* value before the ‘elbow.' (f) The average silhouette value for different cluster numbers. It is a numeric number between 0 and 1, and a high silhouette value implies that the sample is well-suited to its own cluster but weakly related to other clusters. (g) Consensus clustering of the dataset (*k* = 2).

**Figure 2:**
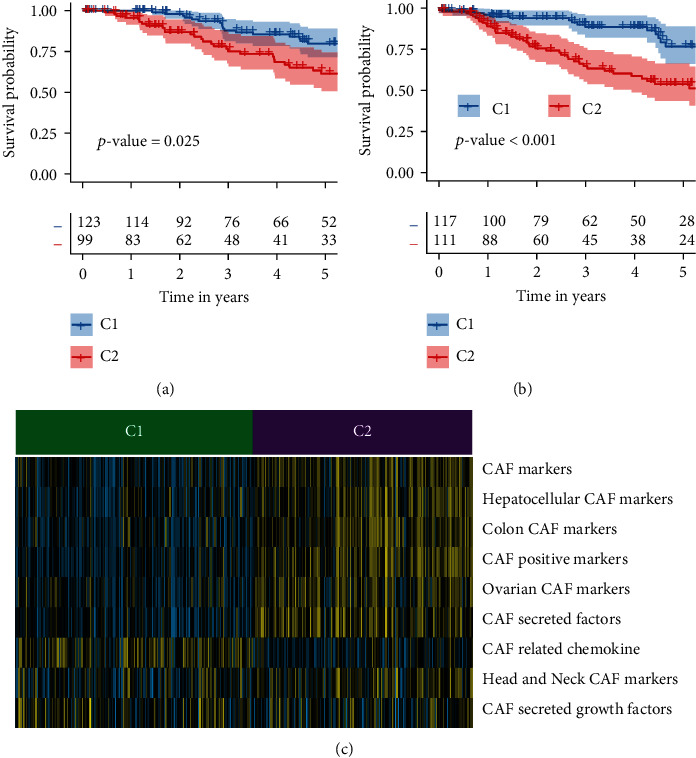
A classification of TNBC patients based on cancer-associated fibroblasts (CAF) subtypes that differ in survival curves and the expression level of CAF gene sets. (a) C1 samples have a better overall survival (OS) profile than C2 samples according to the Kaplan-Meier (K-M) plot. (b) C1 samples have a better progression-free survival (PFS) profile than C2 samples according to the Kaplan-Meier (K-M) plot. In order to determine whether the differences are statistically significant, the log-rank test is performed. (c) In the heatmap, the distribution of expression of the CAF-related gene sets is shown.

GSVA was used to convert the gene-expression matrix of 4 datasets into the matrix of CAF gene sets. Before the conversion, PCA revealed a clear batch effect among these 4 datasets (Figure 1(a)). The batch effect was successfully reduced after the conversion, according to PCA findings (Figure 1(b)). To obtain the accurate CAF subtypes among TNBC samples, we performed CC on the matrix of CAF gene sets. The parameter of clustering numbers from 2 to 6 was selected by the tracking plot, delta area, and CDF results. The results from tracking plot suggested “2” (Figure 1(c)). The CDF plot suggested “4” (Figure 1(d)), and the relative change area under CDF plot suggested “3” (Figure 1(e)). The average silhouette values were used for optimal cluster number selection (Figure 1(f)). It is a numeric number between 0 and 1, and a high silhouette value implies that the sample is well-suited to its own cluster but weakly related to other clusters. The average silhouette values suggested ‘2' (Figure 1(f)). The *p* values from OS and progression-free survival (PFS) analysis if the clustering number was set as “3” (Supplementary Figure 1A) were 0.091 and 0.02 (Supplementary Figure 1B-C). The *p* values from OS and PFS analyses if the clustering number was set as “4” (Supplementary Figure 1D) were 0.23 and 0.043 (Supplementary Figure 1E-F). Thus, the cluster number was finally set as 2 (Figure 1G) because its *p* values (OS: 0.025; PFS: <0.001) in the OS and PFS analyses were significant (Figures 2(a) and 2(b)). Patients in C1 witnessed a significant increase in the OS and PFS time than C2. The proportion of CAF subtypes across different clinical and pathological aspects of TNBC patients was depicted in Table 1. The result indicated that CAF subtypes had no relationships with clinical and pathological parameters such as dataset, age, and stage. Among these two subtypes, C1 had higher levels of PD1 and PDL1 (Supplementary Figure 2).

### 3.2. CAF Subtypes with Distinct CAF and Immune Cells

The levels of CAF gene sets were significantly different between two CAF subtypes (Figure 2(c)). C2 subtypes had significantly higher levels of most CAF gene sets; thus, this subtype was named “CAF^+^” subtype. The C1 was named “CAF^−^ subtype” since it lacked most types of CAF gene sets. Interestingly, unlike other CAF genes sets, the chemokine biomarkers were significantly in CAF- subtype.

**Figure 3:**
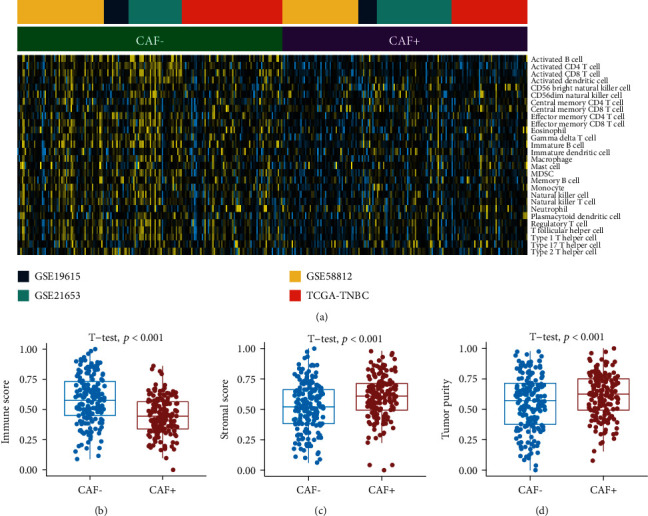
The level of immune cells differs between cancer-associated fibroblast (CAF) subtypes. (a) The heatmap depicts the GSVA-calculated abundance of immune cell populations. (b–d) The box plots show differences in immune score, stromal score, and tumor purity between CAF subtypes based on the GSVA estimation. To compare scores between two groups, the unpaired Student's *t*-test was used. Note: GSVA: gene set variation analysis.

We also explored and compared the immune cells between two CAF subtypes. The CAF- subtype had higher levels of immune cells infiltration (Figure 3(a)). Similarly, CAF- samples had higher immune scores, lower stromal scores, and lower tumor purity, while CAF+ samples had lower immune scores, higher stromal scores, and greater tumor purity (*p* value < 0.001, Student's *t*-test, Figures 3(b)–3(d)).

### 3.3. Analysis of DEGs and Enrichment Analysis

**Figure 4:**
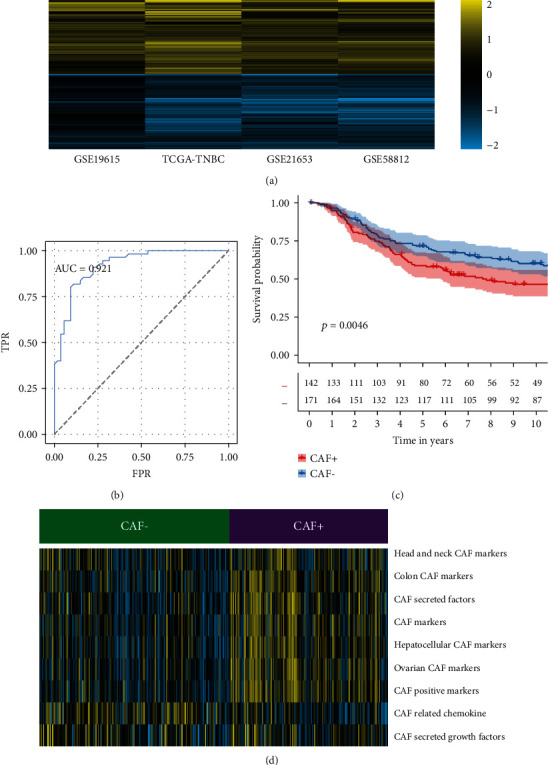
CAF subtypes were validated using independent datasets. (a) The heatmap shows robust DEGs computed using the robust rank aggregation (RRA) algorithm. The yellow color represents the higher log_2_(FoldChange) values and the blue color represents the lower log_2_(FoldChange) values. (b) The AUC value was generated using random forest model on the testing dataset. (c) Compared with CAF+ samples, CAF- samples show a better overall survival (OS) profile in the Kaplan-Meier (K-M) plot from an independent breast cancer dataset (METABRIC dataset). (d) In the heatmap, the distribution of expression of CAF related gene sets from the independent dataset (METABRIC dataset) is shown. Note: AUC: area under the curve.

DEGs were identified between CAF subtypes (*p* value < 0.05 and log2FoldChange > 0.5; Supplementary Figure 3). In CAF+ samples, there were 895 (GSE19615), 649 (GSE21653), 711 (GSE58812), and 890 (TCGA-TNBC) upregulated expressed genes. There were 526 (GSE19615), 848 (GSE21653), 1061 (GSE58812), and 960 (TCGA-TNBC) elevated expressed genes in the CAF- subtype. The RRA approach identified 553 robust DEGs, including 262 upregulated and 291 downregulated genes in the CAF+ subtype. The heatmap was used to visualize the selected robust DEGs (Figure 4(a)).

Enrichment analysis was used to find the enriched pathways associated with 553 robust DEGs. In Supplementary Figure 4, CAF- subtype was largely associated with immune pathways, including ‘leukocyte-activation' (GO), ‘regulation-of-leukocyte-proliferation' (GO), and ‘regulation-of-antigen-receptor' (GO), ‘cytokine-and-cytokine-receptor-interaction' (KEGG), ‘chemokine-signaling-pathway' (KEGG), ‘hematopoietic-cell-lineage' (KEGG), and ‘immunoregulatory-interactions' (REACTOME). On the other hand, the pathways related to extracellular-matrix-organization were found in CAF+ subtype such as ‘TGF-beta-signaling-pathway' (KEGG), ‘focal-adhesion' (KEGG), and ‘ECM-receptor-interaction' (KEGG), ‘degradation-of-the-extracellular-matrix' (REACTOME), and ‘regulation-of-cellular-response-to-growth-factor-stimulus' (REACTOME).

### 3.4. Selection of Genes and Construction of Machine Learning Models

Based on 553 robust DEGs, 59 prognostic-related genes were identified using a univariate Cox regression model. The expression of these genes was used to construct the RF model to predict the CAF subtype. The available TNBC samples were divided into the training (50 percent) and testing datasets (50 percent). Gene expression values were discretized by the median value into discrete values. Based on the parameter search and the fivefold cross-validation procedure in the training dataset, the prediction abilities of machine learning models such as RF, KNN, and DT were evaluated. Among these three machine learning models, RF that showed the highest AUC value was selected. According to the highest values of areas under the curve for the RF model, “mtry =24” was selected (Table 2). In Supplementary Table 2, 9 variables/genes were prioritized and shown according to their importance. The RF model was trained by these 9 genes on the training dataset. An AUC value of 0.921 was obtained in the testing dataset by the constructed RF model (Figure 4(b)).

### 3.5. Predictive Model Validation by an Independent Breast Cancer Dataset

These 9 genes selected for model construction were collagen type X alpha 1 (COL10A1), a disintegrin and metalloproteinase with thrombospondin motifs-12 (ADAMTS12), collagen type XI alpha 1 (COL11A1), endothelin receptor type A (EDNRA), C-X-C motif chemokine receptor 6 (CXCR6), Wnt family member 7B (WNT7B), C-X-C motif chemokine 11 (CXCL11), adipocyte enhancer binding protein 1 (AEBP1), and Epiplakin 1 (EPPK1). These genes were selected as CAF subtype-related genes.

Based on the expression matrix of 9 genes from the METABRIC dataset, the CAF subtype was predicted. A higher prognosis was observed for CAF- subtype samples compared to CAF+ subtype samples (*p* value = 0.0046, Figure 4(c)). The CAF+ subtype samples in the validation dataset had higher levels of CAF gene sets than the CAF- subtype (Figure 4(d)). It is also worth noting that these results were also consistent with the training data (Figures 2(a) and 2(c)).

### 3.6. Investigation of CAF Subtype-Related Genes with Prognosis and CAF Subtypes

**Figure 5:**
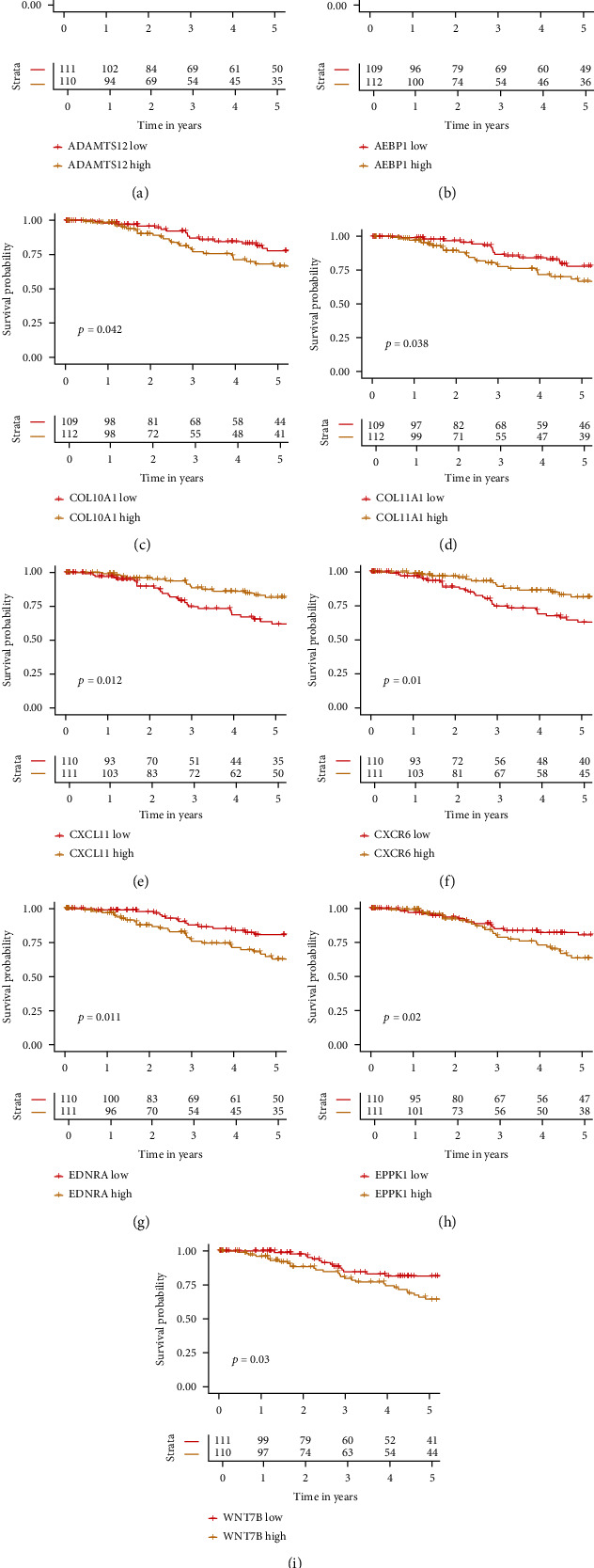
Overall survival (OS) curves for nine CAF subtype-related genes (ADAMTS12, AEBP1, COL10A1, COL11A1, CXCL11, CXCR6, EDNRA, EPPK1, and WNT7B) that are used for model construction. ADAMTS12, AEBP1, COL10A1, COL11A1, EDNRA, EPPK1, and WNT7B were correlated with poor prognosis when their expression values were high. The positive outcome was correlated with the high expression values of CXCL11 and CXCR6.

In the TCGA-TNBC dataset, ADAMTS12, AEBP1, COL10A1, COL11A1, EDNRA, EPPK1, and WNT7B were correlated with poor prognosis when their expression values were high (Figure 5). The positive outcome was correlated with the high expression values of CXCL11 and CXCR6 (Figure 5). For ADAMTS12, AEBP1, COL10A1, COL11A1, CXCL11, EPPK1, and WNT7B, their expression values were higher in tumor samples than in normal samples (Supplementary Figure 5A). For ADAMTS12, AEBP1, COL10A1, COL11A1, EDNRA, EPPK1, and WNT7B, their mRNA expression values were higher in CAF+ samples than CAF- samples (Supplementary Figure 5B). For CXCL11 and CXCR6, their mRNA expression values were higher in CAF- samples than CAF+ samples (Supplementary Figure 5B).

### 3.7. Evaluation of CAF Subtype's Influence on Immunotherapy Response

**Figure 6:**
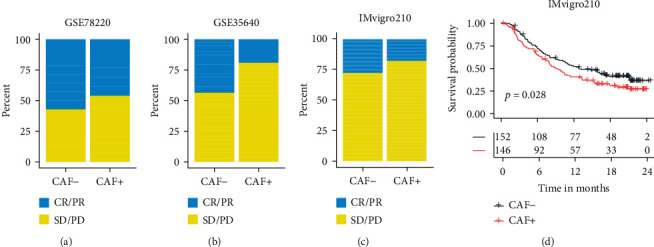
The correlation of predicted cancer-associated fibroblast (CAF) subtype with the immunotherapy efficacy in the independent datasets. (a–c) The association between immunotherapy response rates and CAF subtypes was predicted from independent datasets. (d) In the IMvigor210 dataset, the predicted CAF subtype is correlated with the survival analysis. Note: CR: complete response; PR: partial response; SD: stable disease; PD: progressive disease.

To test the CAF subtype prediction model, three independent datasets (GSE78220, GSE35640, and IMvigor210) containing RNA sequencing data of patients before immunotherapy were chosen to evaluate the CAF subtype's influence on immunotherapy response. GSE78220 contains 28 melanoma samples treated with anti-PD-1 therapy, GSE35640 contains 65 melanoma and lung cancer samples treated with MAGE-A3 immunotherapeutic therapy, and IMvigor210 contains 348 cancer samples treated with anti-PD-L1 therapy. Patients from these cohorts were classified into CAF+ or CAF- subtypes by the expression levels of 9 genes (COL10A1, ADAMTS12, COL11A1, EDNRA, CXCR6, WNT7B, CXCL11, AEBP1, and EPPK1). Within GSE78220 (Figure 6(a)), GSE35640 (Figure 6(b)), and IMvigor210 (Figure 6(c)), the response rates were different by 11%, 24%, and 10%, respectively. There was a greater gain in OS with CAF- than with CAF+ (Figure 6(d)).

### 3.8. Expression Validation for CAF Subtype-Related Genes in Breast Cancer

Among the nine selected genes, protein expression data of ADAMTS12, AEBP1, CXCL11, EDNRA, and EPPK1 were available in the HPA dataset. The IHC score results demonstrated that ADAMTS12, AEBP1, CXCL11, and EPPK1 protein levels were higher in breast cancer samples than in normal controls (Supplementary Figure 6).

## 4. Discussion

Recent studies have found that CAF participates in angiogenesis, tumor cell proliferation, treatment resistance, immunomodulation, and metastases in solid tumors such as breast cancer [37]. However, current research is very limited concerning CAF's role in breast cancer. According to our study, the degree of CAF in TME is greater in patients with the worse prognosis, and it is suggested that CAF is one of the independent prognostic factors. We also estimated the correlation of CAF subtypes with tumor purity, immune cell infiltration, and response rate to ICB. The results suggested that CAF might exert its effect on prognosis by promoting tumor cells and inhibiting immune cells such as CD8 T cells.

Among two CAF subtypes, immune cells were found to be higher in the CAF- subtype than in the CAF+ subtype. Similarly, immune related pathways such as ‘cytokine-cytokine-receptor-interaction,' ‘T-cell-receptor-signaling,' ‘chemokine-signaling,' nad ‘natural-killer-cell-mediated-cytotoxicity' were higher in the CAF- subtype. As a result of these findings, we can assume that CAF is associated with a microenvironment that suppresses immunity. CD8+ T cells could further differentiate into effector cells to kill tumor cells. CAF was reported to suppress CD8+ T cells by PDL2 and FASL [38]. CAF could secrete IL6 that could increase regulatory T cells and decrease CD8+ T cell [39]. In breast cancer, fibroblast activation protein- (FAP-) positive CAF could suppress immune by enhancing the regulatory T cells and inhibiting T cell effectors [40]. Since the tumor-infiltrating T cell is one of the crucial biomarkers for indicating the ICB response [41], the CAF subtypes could also affect the therapeutic efficacy of ICB. Studies show that CAFs decrease sensitivity to anti-PD-L1 treatment [40]. The result from independent ICB datasets also shows that patients in CAF+ subtype have a lower response rate and worse prognosis to ICB. Thus, CAF- subtype patients are the ideal candidates for receiving ICB. Besides, targeting CAF might be a promising therapeutic approach, in complement to conventional treatments and immunotherapies.

Chemokines including CXCL5, CXCL9, CXCL12, CCL3, CCL5, and CXCL16 could be derived from CAF [26, 42]. For example, using western blotting assay and immunofluorescence, CXCL5 expression was high in CAFs [42]. However, the resources of these chemokines are multiple. CXCL5 can be produced by tumor cells, macrophages, and neutrophils [43]. Dendritic cells (DCs) could release CXCL5, CXCL9 and use these chemokines to recruit immune cells such as CD8+ T cells and natural killer cells into the TME [44]. Since the immune cells are found to be inhibited in CAF+ subtype, these results suggest that the CAF is not the main resource of these chemokines.

COL10A1 and COL11A1, as members of the collagen family, are upregulated in breast cancer fibroblasts [45, 46]. ADAMTS12 is a secreted metalloprotease and plays a protumoral role in breast cancer by increasing the capacity for migration and invasion of breast cancer tumor cells [47, 48]. It has been found that inhibiting EDNRA could inhibit the invasion of BC tumor cells [49]. WNT7B is one of the Wnt pathway proteins, and clinical outcome of BC patients with high expression of WNT7B is poor [50]. AEBP1 is one of the transcriptional repressors that could improve BC progression through extracellular matrix thickening [51]. EPPK1 is part of the epidermal growth factor (EGF) signal and is found to promote the proliferation of tumor cells [52]. CXCR6 and CXCL11 are members of chemokines, and CXCR6 is required for antitumor efficacy of CD8+ T cell infiltration [53, 54]. However, another study found that CXCR6 could increase cell migration, invasion, and metastasis of breast cancer [55]. This phenomenon might be caused by the diverse origins of chemokines, and more studies are needed to clarify their roles in TNBC.

The study has some limitations. Firstly, we only used pure bioinformatics techniques to predict CAF in TME. In order to ensure the robustness of our findings, we selected multiple independent datasets. Secondly, there are no specific biomarkers for CAF because of the high heterogeneity of CAF origin, phenotype, and function [56]. The biomarkers of distinct CAF subgroups may be different, even opposite. Lastly, the differences among CAFs were overlooked in our study.

## 5. Conclusion

CAF is linked to lower survival rates for TNBC patients and suppressed immune activity. In summary, CAF could lead to the decreased ICB response rate. Simultaneously, the random forest model composed of COL10A1, ADAMTS12, COL11A1, EDNRA, CXCR6, WNT7B, CXCL11, AEBP1, and EPPK1 is a promising tool for the prediction of the CAF subtype.

## Figures and Tables

**Table 1:** Clinical characteristics of CAF subtypes.

Characteristics	C1 (CAF-)	C2 (CAF+)	*p* value
	*n* = 174 (100%)	*n* = 161 (100%)	
Datasets			0.167
GSE19615	16 (9.20%)	12 (7.45%)	
GSE21653	35 (20.1%)	49 (30.4%)	
GSE58812	57 (32.8%)	50 (31.1%)	
TCGA-TNBC	66 (37.9%)	50 (31.1%)	
Age (years)			0.569
20-50	57 (32.8%)	50 (31.1%)	
50-70	94 (54.0%)	83 (51.6%)	
70-90	23 (13.2%)	28 (17.4%)	
Stage			0.708
Stage I-II	78 (44.8%)	75 (46.6%)	
Stage III-IV	21 (12.1%)	23 (14.3%)	
Not available	75 (43.1%)	63 (39.1%)	

**Table 2:** The parameter selection in machine learning models.

Parameter	ROC	Sens	Spec
mtry:52	0.926	0.839	0.790
mtry:40	0.924	0.839	0.803
mtry:35	0.922	0.817	0.790
Cp:0.0	0.862	0.758	0.827
Cp:0.134	0.774	0.771	0.777
Cp:0.202	0.774	0.771	0.777
K:21	0.897	0.737	0.815
K:19	0.897	0.703	0.827
K:17	0.896	0.692	0.815

ROC: receiver operating characteristic; Sens: sensitivity; Spec: specificity.

## Data Availability

The datasets were downloaded from the TCGA database (https://tcga-data.nci.nih.gov/tcga/) and the GEO database (http://www.ncbi.nlm.nih.gov/geo/).

## Supplementary Materials

Supplementary 1 Supplementary Figure 1: cluster analysis and survival curves of clusters. (A) An illustration of the consensus matrix at *k* = 3 is shown in the heatmap. (B) Survival analysis (OS) of patients with the three subtypes. (C) Survival analysis (PFS) of patients with the three subtypes. (D) An illustration of the consensus matrix at *k* = 4 is shown in the heatmap. (E) Survival analysis (OS) of patients with the four subtypes. (F) Survival analysis (PFS) of patients with the four subtypes. The log-rank test was conducted to determine the significance of the differences among subtypes. Note: OS: overall survival; PFS: progression-free survival. Supplementary Figure 2: the mRNA expression values of PD1 and PDL1 between two CAF subtypes. (A) Programmed cell death protein 1 (PD1). (B) Programmed death-ligand 1 (PDL1). Supplementary Figure 3: volcano plots for DEGs. (A–D) Volcano plots for differentially expressed genes in GSE19615, GSE21653, GSE58812, and TCGA-TNBC. Upregulated genes and downregulated genes in CAF+ samples are represented by the red and blue points, respectively. Supplementary Figure 4: enrichment analysis of robust differentially expressed genes (DEGs). Note: NES: normalized enrichment score. Supplementary Figure 5: the expression pattern of CAF subtype-related genes (ADAMTS12, AEBP1, COL10A1, COL11A1, CXCL11, CXCR6, EDNRA, EPPK1, and WNT7B). (A) The mRNA expression values of CAF subtype-related genes between normal and tumor samples. (B) The mRNA expression values of CAF subtype-related genes between CAF+ and CAF- samples. Supplementary Figure 6: protein expression values of ADAMTS12, AEBP1, CXCL11, EDNRA, and EPPK1. Representative immunohistochemistry (IHC) images of ADAMTS12 (A), AEBP1 (C), CXCL11 (E), EDNRA (G), and EPPK1 (I) in normal (left) and breast cancer (right) tissues in the Human Protein Atlas (HPA) dataset. The difference of IHC scores of ADAMTS12 (B), AEBP1 (D), CXCL11 (F), EDNRA (H), and EPPK1 (J) in normal and breast cancer tissues in the Human Protein Atlas (HPA) dataset.

Supplementary 2 Supplementary Table 1: commonly used CAF markers. Supplementary Table 2: the importance of variables in random forest model.
