# Supplementary figures and images for: Identification of Cancer-Associated Fibroblast Subtype of Triple-Negative Breast Cancer
Source: J Oncol. 2022 Apr 23;2022:6452636. doi: 10.1155/2022/6452636 (PMC9057104; doi:10.1155/2022/6452636)

**A**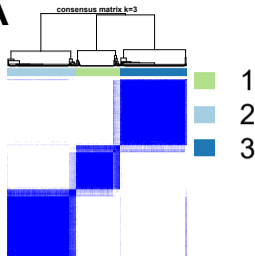**B**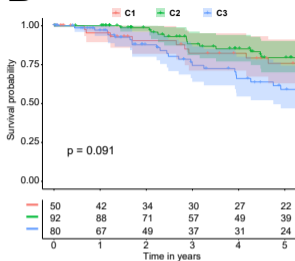**C**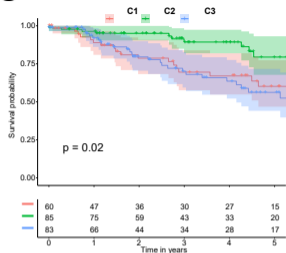**D**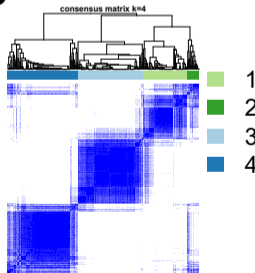**E**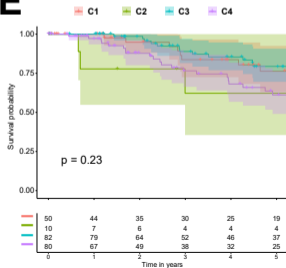**F**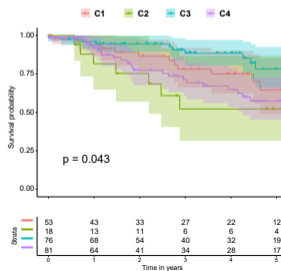

Supplement: Supplementary 1 — Supplementary Figure 1: cluster analysis and survival curves of clusters. (A) An illustration of the consensus matrix at k = 3 is shown in the heatmap. (B) Survival analysis (OS) of patients with the three subtypes. (C) Survival analysis (PFS) of patients with the three subtypes. (D) An illustration of the consensus matrix at k = 4 is shown in the heatmap. (E) Survival analysis (OS) of patients with the four subtypes. (F) Survival analysis (PFS) of patients with the four subtypes. The log-rank test was conducted to determine the significance of the differences among subtypes. Note: OS: overall survival; PFS: progression-free survival. Supplementary Figure 2: the mRNA expression values of PD1 and PDL1 between two CAF subtypes. (A) Programmed cell death protein 1 (PD1). (B) Programmed death-ligand 1 (PDL1). Supplementary Figure 3: volcano plots for DEGs. (A–D) Volcano plots for differentially expressed genes in GSE19615, GSE21653, GSE58812, and TCGA-TNBC. Upregulated genes and downregulated genes in CAF+ samples are represented by the red and blue points, respectively. Supplementary Figure 4: enrichment analysis of robust differentially expressed genes (DEGs). Note: NES: normalized enrichment score. Supplementary Figure 5: the expression pattern of CAF subtype-related genes (ADAMTS12, AEBP1, COL10A1, COL11A1, CXCL11, CXCR6, EDNRA, EPPK1, and WNT7B). (A) The mRNA expression values of CAF subtype-related genes between normal and tumor samples. (B) The mRNA expression values of CAF subtype-related genes between CAF+ and CAF- samples. Supplementary Figure 6: protein expression values of ADAMTS12, AEBP1, CXCL11, EDNRA, and EPPK1. Representative immunohistochemistry (IHC) images of ADAMTS12 (A), AEBP1 (C), CXCL11 (E), EDNRA (G), and EPPK1 (I) in normal (left) and breast cancer (right) tissues in the Human Protein Atlas (HPA) dataset. The difference of IHC scores of ADAMTS12 (B), AEBP1 (D), CXCL11 (F), EDNRA (H), and EPPK1 (J) in normal and breast cancer tissues in the Human [file 6452636.f1.zip › Supplementary Figure 1 (3).pdf]

C1

C2

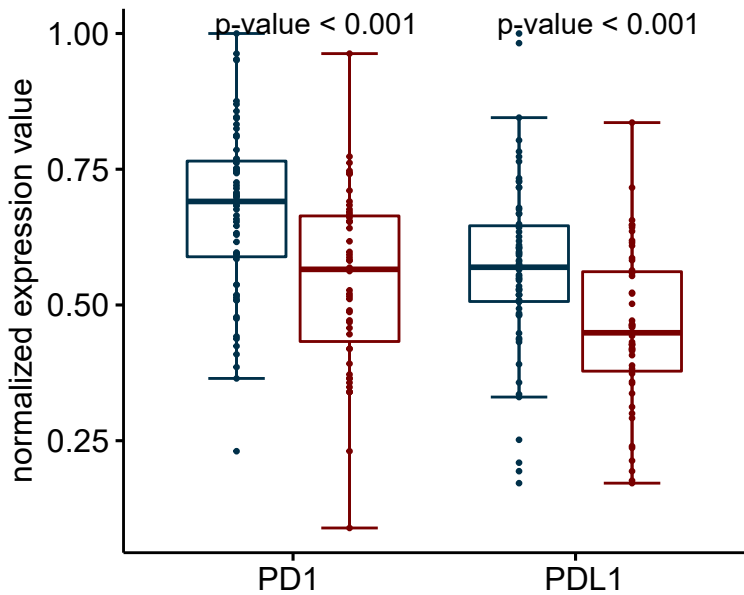

Supplement: Supplementary 1 — Supplementary Figure 1: cluster analysis and survival curves of clusters. (A) An illustration of the consensus matrix at k = 3 is shown in the heatmap. (B) Survival analysis (OS) of patients with the three subtypes. (C) Survival analysis (PFS) of patients with the three subtypes. (D) An illustration of the consensus matrix at k = 4 is shown in the heatmap. (E) Survival analysis (OS) of patients with the four subtypes. (F) Survival analysis (PFS) of patients with the four subtypes. The log-rank test was conducted to determine the significance of the differences among subtypes. Note: OS: overall survival; PFS: progression-free survival. Supplementary Figure 2: the mRNA expression values of PD1 and PDL1 between two CAF subtypes. (A) Programmed cell death protein 1 (PD1). (B) Programmed death-ligand 1 (PDL1). Supplementary Figure 3: volcano plots for DEGs. (A–D) Volcano plots for differentially expressed genes in GSE19615, GSE21653, GSE58812, and TCGA-TNBC. Upregulated genes and downregulated genes in CAF+ samples are represented by the red and blue points, respectively. Supplementary Figure 4: enrichment analysis of robust differentially expressed genes (DEGs). Note: NES: normalized enrichment score. Supplementary Figure 5: the expression pattern of CAF subtype-related genes (ADAMTS12, AEBP1, COL10A1, COL11A1, CXCL11, CXCR6, EDNRA, EPPK1, and WNT7B). (A) The mRNA expression values of CAF subtype-related genes between normal and tumor samples. (B) The mRNA expression values of CAF subtype-related genes between CAF+ and CAF- samples. Supplementary Figure 6: protein expression values of ADAMTS12, AEBP1, CXCL11, EDNRA, and EPPK1. Representative immunohistochemistry (IHC) images of ADAMTS12 (A), AEBP1 (C), CXCL11 (E), EDNRA (G), and EPPK1 (I) in normal (left) and breast cancer (right) tissues in the Human Protein Atlas (HPA) dataset. The difference of IHC scores of ADAMTS12 (B), AEBP1 (D), CXCL11 (F), EDNRA (H), and EPPK1 (J) in normal and breast cancer tissues in the Human [file 6452636.f1.zip › Supplementary Figure 2 (3).pdf]

**A**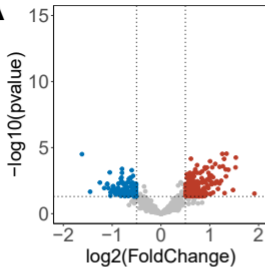**B**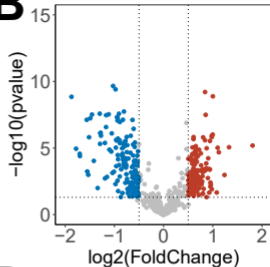**C**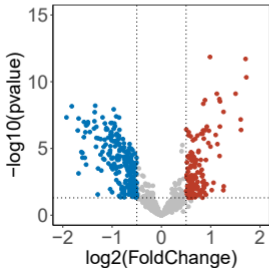**D**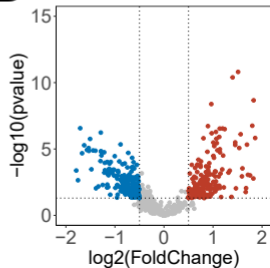

Supplement: Supplementary 1 — Supplementary Figure 1: cluster analysis and survival curves of clusters. (A) An illustration of the consensus matrix at k = 3 is shown in the heatmap. (B) Survival analysis (OS) of patients with the three subtypes. (C) Survival analysis (PFS) of patients with the three subtypes. (D) An illustration of the consensus matrix at k = 4 is shown in the heatmap. (E) Survival analysis (OS) of patients with the four subtypes. (F) Survival analysis (PFS) of patients with the four subtypes. The log-rank test was conducted to determine the significance of the differences among subtypes. Note: OS: overall survival; PFS: progression-free survival. Supplementary Figure 2: the mRNA expression values of PD1 and PDL1 between two CAF subtypes. (A) Programmed cell death protein 1 (PD1). (B) Programmed death-ligand 1 (PDL1). Supplementary Figure 3: volcano plots for DEGs. (A–D) Volcano plots for differentially expressed genes in GSE19615, GSE21653, GSE58812, and TCGA-TNBC. Upregulated genes and downregulated genes in CAF+ samples are represented by the red and blue points, respectively. Supplementary Figure 4: enrichment analysis of robust differentially expressed genes (DEGs). Note: NES: normalized enrichment score. Supplementary Figure 5: the expression pattern of CAF subtype-related genes (ADAMTS12, AEBP1, COL10A1, COL11A1, CXCL11, CXCR6, EDNRA, EPPK1, and WNT7B). (A) The mRNA expression values of CAF subtype-related genes between normal and tumor samples. (B) The mRNA expression values of CAF subtype-related genes between CAF+ and CAF- samples. Supplementary Figure 6: protein expression values of ADAMTS12, AEBP1, CXCL11, EDNRA, and EPPK1. Representative immunohistochemistry (IHC) images of ADAMTS12 (A), AEBP1 (C), CXCL11 (E), EDNRA (G), and EPPK1 (I) in normal (left) and breast cancer (right) tissues in the Human Protein Atlas (HPA) dataset. The difference of IHC scores of ADAMTS12 (B), AEBP1 (D), CXCL11 (F), EDNRA (H), and EPPK1 (J) in normal and breast cancer tissues in the Human [file 6452636.f1.zip › Supplementary Figure 3 (3).pdf]

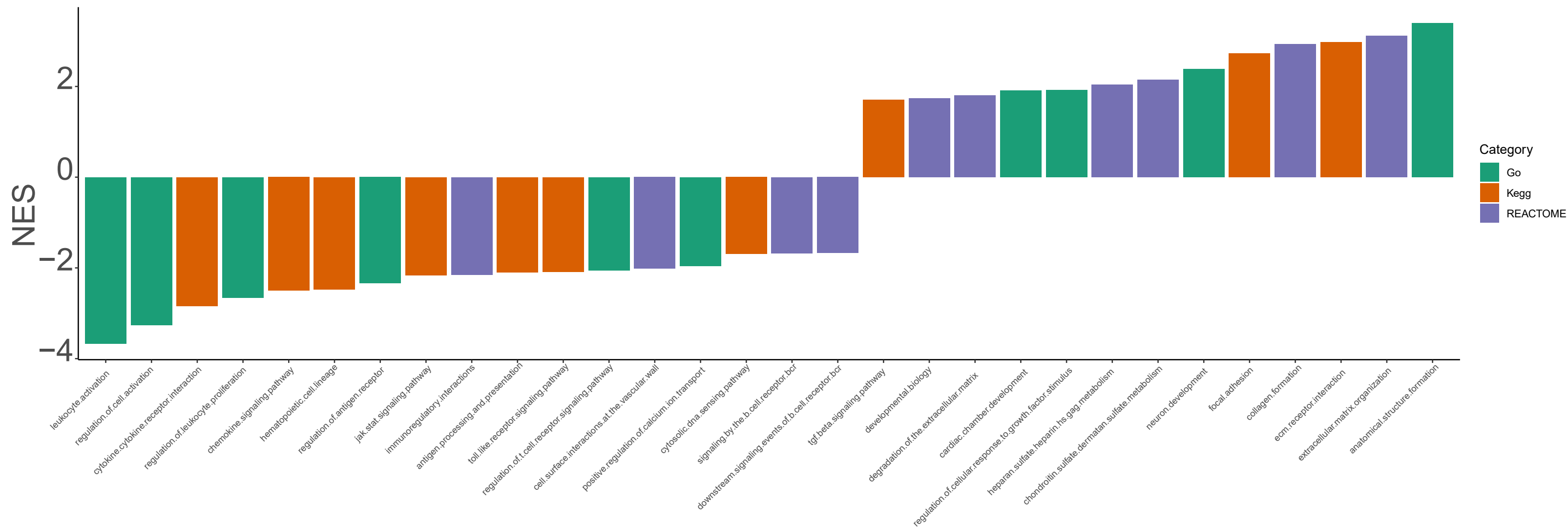

Supplement: Supplementary 1 — Supplementary Figure 1: cluster analysis and survival curves of clusters. (A) An illustration of the consensus matrix at k = 3 is shown in the heatmap. (B) Survival analysis (OS) of patients with the three subtypes. (C) Survival analysis (PFS) of patients with the three subtypes. (D) An illustration of the consensus matrix at k = 4 is shown in the heatmap. (E) Survival analysis (OS) of patients with the four subtypes. (F) Survival analysis (PFS) of patients with the four subtypes. The log-rank test was conducted to determine the significance of the differences among subtypes. Note: OS: overall survival; PFS: progression-free survival. Supplementary Figure 2: the mRNA expression values of PD1 and PDL1 between two CAF subtypes. (A) Programmed cell death protein 1 (PD1). (B) Programmed death-ligand 1 (PDL1). Supplementary Figure 3: volcano plots for DEGs. (A–D) Volcano plots for differentially expressed genes in GSE19615, GSE21653, GSE58812, and TCGA-TNBC. Upregulated genes and downregulated genes in CAF+ samples are represented by the red and blue points, respectively. Supplementary Figure 4: enrichment analysis of robust differentially expressed genes (DEGs). Note: NES: normalized enrichment score. Supplementary Figure 5: the expression pattern of CAF subtype-related genes (ADAMTS12, AEBP1, COL10A1, COL11A1, CXCL11, CXCR6, EDNRA, EPPK1, and WNT7B). (A) The mRNA expression values of CAF subtype-related genes between normal and tumor samples. (B) The mRNA expression values of CAF subtype-related genes between CAF+ and CAF- samples. Supplementary Figure 6: protein expression values of ADAMTS12, AEBP1, CXCL11, EDNRA, and EPPK1. Representative immunohistochemistry (IHC) images of ADAMTS12 (A), AEBP1 (C), CXCL11 (E), EDNRA (G), and EPPK1 (I) in normal (left) and breast cancer (right) tissues in the Human Protein Atlas (HPA) dataset. The difference of IHC scores of ADAMTS12 (B), AEBP1 (D), CXCL11 (F), EDNRA (H), and EPPK1 (J) in normal and breast cancer tissues in the Human [file 6452636.f1.zip › Supplementary Figure 4 (3).pdf]

**A**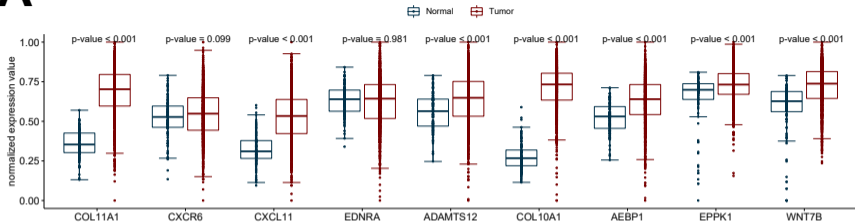**B**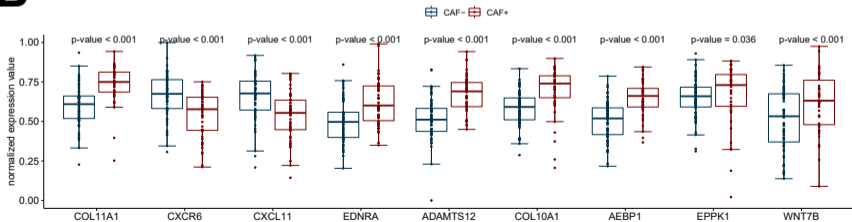

Supplement: Supplementary 1 — Supplementary Figure 1: cluster analysis and survival curves of clusters. (A) An illustration of the consensus matrix at k = 3 is shown in the heatmap. (B) Survival analysis (OS) of patients with the three subtypes. (C) Survival analysis (PFS) of patients with the three subtypes. (D) An illustration of the consensus matrix at k = 4 is shown in the heatmap. (E) Survival analysis (OS) of patients with the four subtypes. (F) Survival analysis (PFS) of patients with the four subtypes. The log-rank test was conducted to determine the significance of the differences among subtypes. Note: OS: overall survival; PFS: progression-free survival. Supplementary Figure 2: the mRNA expression values of PD1 and PDL1 between two CAF subtypes. (A) Programmed cell death protein 1 (PD1). (B) Programmed death-ligand 1 (PDL1). Supplementary Figure 3: volcano plots for DEGs. (A–D) Volcano plots for differentially expressed genes in GSE19615, GSE21653, GSE58812, and TCGA-TNBC. Upregulated genes and downregulated genes in CAF+ samples are represented by the red and blue points, respectively. Supplementary Figure 4: enrichment analysis of robust differentially expressed genes (DEGs). Note: NES: normalized enrichment score. Supplementary Figure 5: the expression pattern of CAF subtype-related genes (ADAMTS12, AEBP1, COL10A1, COL11A1, CXCL11, CXCR6, EDNRA, EPPK1, and WNT7B). (A) The mRNA expression values of CAF subtype-related genes between normal and tumor samples. (B) The mRNA expression values of CAF subtype-related genes between CAF+ and CAF- samples. Supplementary Figure 6: protein expression values of ADAMTS12, AEBP1, CXCL11, EDNRA, and EPPK1. Representative immunohistochemistry (IHC) images of ADAMTS12 (A), AEBP1 (C), CXCL11 (E), EDNRA (G), and EPPK1 (I) in normal (left) and breast cancer (right) tissues in the Human Protein Atlas (HPA) dataset. The difference of IHC scores of ADAMTS12 (B), AEBP1 (D), CXCL11 (F), EDNRA (H), and EPPK1 (J) in normal and breast cancer tissues in the Human [file 6452636.f1.zip › Supplementary Figure 5 (2).pdf]

**A**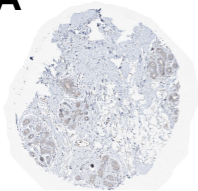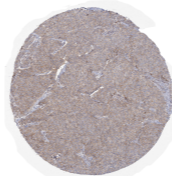**C**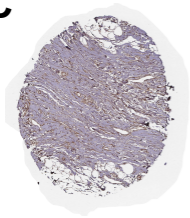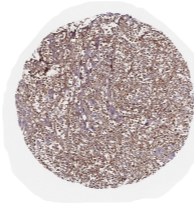**E**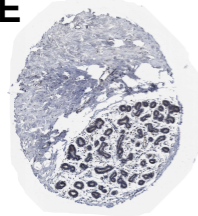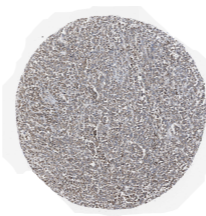**G**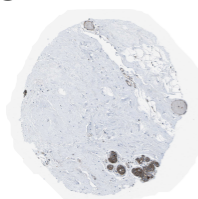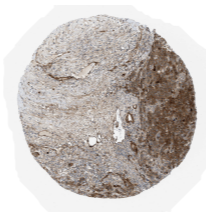**I**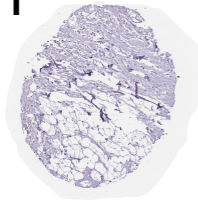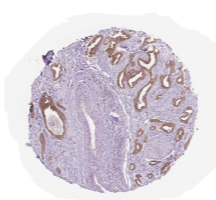**B**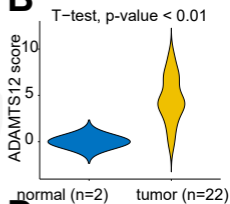**D**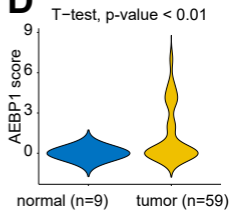**F**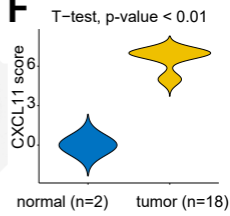**H**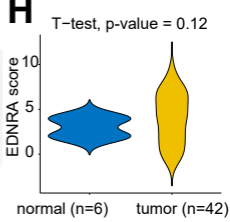**J**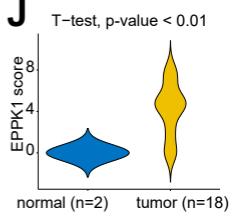

Supplement: Supplementary 1 — Supplementary Figure 1: cluster analysis and survival curves of clusters. (A) An illustration of the consensus matrix at k = 3 is shown in the heatmap. (B) Survival analysis (OS) of patients with the three subtypes. (C) Survival analysis (PFS) of patients with the three subtypes. (D) An illustration of the consensus matrix at k = 4 is shown in the heatmap. (E) Survival analysis (OS) of patients with the four subtypes. (F) Survival analysis (PFS) of patients with the four subtypes. The log-rank test was conducted to determine the significance of the differences among subtypes. Note: OS: overall survival; PFS: progression-free survival. Supplementary Figure 2: the mRNA expression values of PD1 and PDL1 between two CAF subtypes. (A) Programmed cell death protein 1 (PD1). (B) Programmed death-ligand 1 (PDL1). Supplementary Figure 3: volcano plots for DEGs. (A–D) Volcano plots for differentially expressed genes in GSE19615, GSE21653, GSE58812, and TCGA-TNBC. Upregulated genes and downregulated genes in CAF+ samples are represented by the red and blue points, respectively. Supplementary Figure 4: enrichment analysis of robust differentially expressed genes (DEGs). Note: NES: normalized enrichment score. Supplementary Figure 5: the expression pattern of CAF subtype-related genes (ADAMTS12, AEBP1, COL10A1, COL11A1, CXCL11, CXCR6, EDNRA, EPPK1, and WNT7B). (A) The mRNA expression values of CAF subtype-related genes between normal and tumor samples. (B) The mRNA expression values of CAF subtype-related genes between CAF+ and CAF- samples. Supplementary Figure 6: protein expression values of ADAMTS12, AEBP1, CXCL11, EDNRA, and EPPK1. Representative immunohistochemistry (IHC) images of ADAMTS12 (A), AEBP1 (C), CXCL11 (E), EDNRA (G), and EPPK1 (I) in normal (left) and breast cancer (right) tissues in the Human Protein Atlas (HPA) dataset. The difference of IHC scores of ADAMTS12 (B), AEBP1 (D), CXCL11 (F), EDNRA (H), and EPPK1 (J) in normal and breast cancer tissues in the Human [file 6452636.f1.zip › Supplementary Figure 6 (2).pdf]
