# Supplementary material for: Identification of Cancer-Associated Fibroblast Subtype of Triple-Negative Breast Cancer
Source: J Oncol. 2022 Apr 23;2022:6452636. doi: 10.1155/2022/6452636 (PMC9057104; doi:10.1155/2022/6452636)
Supplement: Supplementary 2 — Supplementary Table 1: commonly used CAF markers. Supplementary Table 2: the importance of variables in random forest model. [file 6452636.f2.docx]

| **CAF Gene Sets** | **Genes** |
| --- | --- |
| CAF Markers | ACTA2, FAP, S100A4, PDGFRA, PDGFRB, PDPN, CD90, COL1A1 |
| Hepatocellular CAF | CD90, CD73, CD105, CD29, CD44, CD166, FN, ACTA2, FAP, VIM, COL1A1, PDGFRA, FN1 |
| Colon CAF | MMP2, DCN, COL1A2, ACTA2, PDGFA, TAGLN |
| Head and Neck CAF | CTHRC1, COL1A1, POSTN, TPM4, CFD, APOD, CXCL12, GPC3 |
| Ovarian CAF | FAP, TGF-β, COL11A1, SULF1, IL6, CXCL12 |
| CAF Related Chemokine | CXCL5, CXCL9, CXCL12, CCL3, CCL5, CXCL16, CXCL12 |
| CAF Related Growth Factors | FGF1, FGF2, FGF9, GPER, GDF15, HGF, IGF1, IGF2, TGFB1, VCAM1 |
| CAF Related Factors | ADAM17, ANXA3, CD9, GAS6, CDH11, COL1A1, HIAR, GREM1, LDHA, HMGB1, LOX, LOXL2, POSTN, RANKL, SNAI1, TNFSF4, WNT2 |
| CAF Positive Markers | FAP, ACTA2, MFAP5, COL11A1, TNC, PDPN, ITGA11, NG2 |

**Supplementary Table 1.** Commonly used CAF markers.

| **Gene** | **Importance** |
| --- | --- |
| COL10A1 | 7.63129420 |
| ADAMTS12 | 7.55824861 |
| COL11A1 | 5.54500439 |
| EDNRA | 1.87912874 |
| CXCR6 | 1.74164436 |
| WNT7B | 1.60309494 |
| CXCL11 | 1.49544501 |
| AEBP1 | 1.49191427 |
| EPPK1 | 1.45436398 |

**Supplementary Table 2.** The importance of variables in random forest model.
